# *π*Force—Repeatability and Reliability of Peak Force and Rate of Force Development in a Portable Multi-Exercise Device

**DOI:** 10.3390/muscles4030036

**Published:** 2025-09-01

**Authors:** Ricardo Pimenta, Abel Pimenta

**Affiliations:** 1 Research Center in Sports Sciences, Health Sciences and Human Development (CIDESD), University of Maia, Av. Carlos de Oliveira Campos, Castêlo da Maia, 4475–690 Maia, Portugal; 2 Research Center of the Polytechnic Institute of Maia (N2i), Maia Polytechnic Institute (IPMAIA), Av. Carlos de Oliveira Campos, Castêlo da Maia, 4475–690 Maia, Portugal; 3 Department of Rehabilitation and Optimization of Performance (DROP), Futebol Clube Famalicão–Futebol SAD, Rua da Academia F.C. Famalicão 300, Esmeriz, 4760-482 Famalicão, Portugal; 4 Porto Biomechanics Laboratory, Faculty of Sport, University of Porto, R. Dr. Plácido da Costa 91, 4200-450 Porto, Portugal; 5 Independent Researcher, Porto, Portugal

**Keywords:** muscle strength, knee flexors, isometric mid-thigh pull, seated calf raise

## Abstract

Isometric strength is widely used to monitor training adaptations, assess neuromuscular fatigue, and play a critical role in the maintenance of muscle health. This study assessed repeatability (intra-session) and reliability (inter-session) of a force production machine in different exercises: Isometric Mid-Thigh Pull (IMTP), Knee Flexion (KF) at 30°, and Seated Calf Raise (SCR). Parameters measured included Peak Force (PF), RFD at 0–50, 0–100, 0–150, 0–200, 50–100, 100–150 and 150–200 ms. Thirty male individuals (IMTP = 30; KF = 11 and SCR = 30) participated (age: 20.6 ± 3.6 years, mass: 75.3 ± 7.5 kg, height: 1.80 ± 0.64 m). Repeatability and reliability were calculated for bilateral PF and RFD for IMTP and SCR, and unilateral for KF. PF demonstrated good to excellent repeatability in all exercises: IMTP (ICC = 0.93), KF (left: ICC = 0.98; right: ICC = 0.97), SCR (ICC = 0.84). RFD displayed poor to good repeatability in IMTP (ICC = 0.45–0.87) and SCR (ICC = 0.40–0.85), moderate to excellent repeatability in KF (left: ICC = 0.53–0.96; right: ICC = 0.61–0.92). PF reliability was excellent in IMTP (ICC = 0.93) and KF (left: ICC = 0.99; right: ICC = 0.97), and moderate in SCR (ICC = 0.64). RFD reliability was moderate to excellent in IMTP (ICC = 0.58–0.94), poor to good in SCR (ICC = 0.13–0.64), and poor to excellent in KF (left: ICC = 0.33–0.96; right: ICC = −0.19–0.95). This study shows that portable dynamometry can measure maximal and explosive strength in different exercises, demonstrating good reliability for most parameters in IMTP and KF.

## 1. Introduction

Isometric strength has been used by sports professionals to monitor improvements in strength following training, to understand the neuromuscular fatigue of athletes [1,2], which allows for better understanding of skeletal muscle adaptations to exercise-induced stress [3,4] and play a critical role in the maintenance of muscle health [5]. Fatigue is defined as the inability to sustain a specific exercise intensity or power output, which emerges during and immediately following physical activity, being attributed to a combination of central and peripheral fatigue mechanisms [6]. Central fatigue reduces voluntary muscle activation and predominantly occurs after submaximal, low-intensity muscle contractions [7]. Additionally, peripheral fatigue involves a diminished capacity for muscle contraction and can result from disruptions in action potential propagation, excitation-contraction coupling, and mechanisms of contractile force production [7]. Several exercises are commonly used to assess lower limb strength and training-induced fatigue, particularly in recurring isometric strength assessments. These include the Isometric Mid-Thigh Pull (IMTP) [8,9], Knee Flexion (KF) [4,10], and Seated Calf Raise (SCR) [11]. Maximal force, typically measured as peak force (PF), and the rate of force development (RFD) are key variables evaluated during these exercises to quantify muscle performance and fatigue.

The IMTP is widely recognized as one of the most frequently applied exercises in the field of sports science for the assessment of maximal strength and neuromuscular fatigue in athletes across different sports [2,9,12]. This test has gained considerable popularity not only because of its relatively simple execution and safety, but also due to its strong association with various indicators of dynamic exercise performance [13] which it is more advantageous than isolated single-joint isometric strength tests. This makes it particularly valuable for both researchers and practitioners who aim to monitor performance adaptations or fatigue levels in athletes. A systematic review indicated that sixteen studies reported reliability of absolute PF on IMTP with an intraclass correlation coefficient (ICC) between 0.84 and 0.99 (median ICC = 0.97), with 88% of the ICCs being ≥0.90 [14].

In contrast, regarding the SCR test, no published data is currently available on the reliability or repeatability of this assessment. Nevertheless, some insight can be drawn from prior investigations that examined plantar flexion performance using isokinetic dynamometers, particularly when the knee joint was maintained in an extended position under different testing conditions [15,16]. These studies consistently demonstrated excellent reliability for Peak Torque (PT) during repeated measures within the same session (intrasession). Furthermore, when considering inter-day reliability, the results were generally good to excellent for peak torque and force, but poor for peak RTD (ICC = 0.13) [17]. Moreover, using a hand-held dynamometer to assess plantar flexion peak force, results were even less favorable. Specifically, intra-day reliability was classified as poor to moderate (ICC = 0.56 [0.29–0.74]). Furthermore, inter-tester reliability assessed across different days was weak (ICC = 0.23 and 0.15), indicating substantial variability and inconsistency between assessors. These findings highlight the limitations of certain traditional tools when applied to repeated or field-based strength assessments.

Regarding the KF exercise, previous studies analyzed KF using isokinetic dynamometers [18,19]. These devices are widely regarded as the gold standard for the assessment of joint torque and muscle strength due to their precision and ability to provide controlled testing conditions. Despite their advantages, isokinetic dynamometers also present several important limitations: they are expensive, non-portable, and require significant time and expertise to operate. These factors reduce their practicality for large-scale testing or for use in applied sport settings where quick, accessible, and cost-efficient assessments are needed. To overcome such limitations, a new device that allows easy measurement of athletes’ performance (measuring PF and RFD) and can be used in different contexts is introduced.

Therefore, the primary aim of the present study was to evaluate the test–retest reliability of PF and RFD during three different isometric strength assessments—IMTP, KF, and SCR—when using a portable dynamometer. The primary hypothesis underpinning this investigation was that the portable dynamometer would yield reliable and reproducible measurements of both PF and RFD across all three testing conditions, thereby providing a valid alternative to more costly and less accessible laboratory-based instruments.

## 2. Methods

Thirty physically active males were invited to participate in the study (age: 20.6 ± 3.2 years, body mass: 75.3 ± 7.1 kg, height: 1.79 ± 0.27 m). The sessions for each test were conducted individually, totaling six sessions for the test–retest calculation (2 IMTP, 2 SCR, 2 KF). For the KF only 11 individuals completed the 2 sessions (age: 24.6 ± 3.5 years, body mass: 79.9 ± 6.8 kg, height: 1.81 ± 0.43 m). Therefore, the sample size was IMTP = 30, KF = 11, and SCR = 30. All participants read and signed an informed consent form prior to participating in the study which is in accordance with the Helsinki Declaration. The Ethical Committee at the Faculty of Sports at the University of Porto approved the study (#25/2022).

### 2.1. Protocol

Participants visited the testing facility for an experimental familiarization session and on two occasions for each exercise (IMTP, KF and SCR). The test assessment sessions were separated by 5–7 days. Testing began with a standardized warm-up protocol previously reported [20]. Then, individuals performed 5 submaximal contractions to prepare for the maximum voluntary isometric contraction (MVIC) evaluation. During each exercise assessment, individuals performed 2 MVCs.

### 2.2. Dynamometry

**Figure 1:**
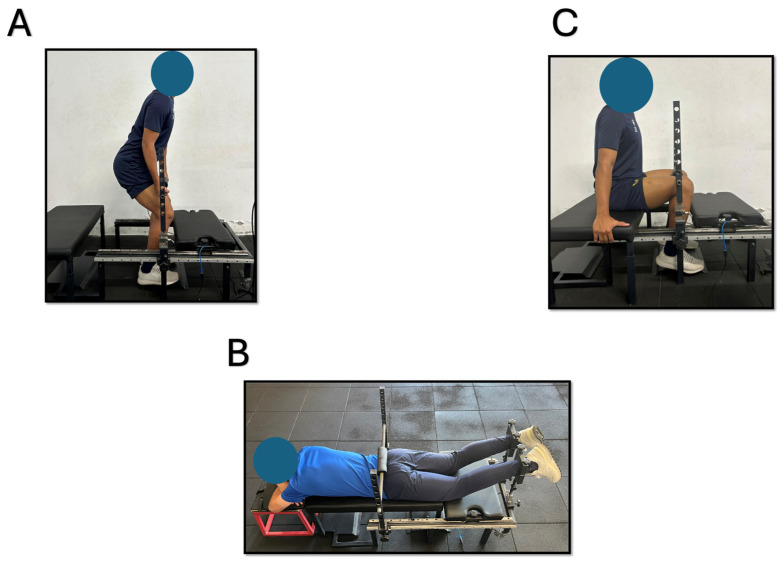
Experimental setup to assess Peak Force and Rate of Force Development in (**A**) Isometric Mid-Thigh Pull–bar height was adjusted up or down to allow the athlete to obtain the optimal knee (125–145°) and hip (140–150°), (**B**) Knee flexion–hip at neutral position and knee flexed at 30° (0° = full extension), and (**C**) Seated Calf Raise–knee flexed at 90°.

The πforce has two load cells (CATLT DYLY103) of 200 kg, each with a maximum capacity of 1961N (one on each side to measure the force applied by each leg). The force was measured at a sampling rate of 80 Hz using an Arduino Uno with an HX711 converter. Participants were placed in standing (IMTP, Figure 1A), prone (KF, Figure 1B), and seated positions (SCR, Figure 1C). For the IMTP, the bar height was adjusted up or down to allow the athlete to obtain the optimal knee (125–145°) and hip (140–150°) angles [21]. For the KF exercise, participants were positioned with the hips in neutral anatomical position and the knees flexed at 30° (0° = full extension) [4]. The distance between the force transducer center and the femoral lateral condyle were measured to estimate the knee torque. Therefore, KF results are expressed in torque as Peak Torque (PT) and Rate of Torque Development (RTD). For the SCR, participants were positioned with the knee flexed at 90°.

Participants were instructed to exert force “as fast and strong as possible” to obtain both PF/PT and RFD/RTD [22]. The PF/PT and RFD/RTD were calculated with Python code (Supplementary Data) using Visual Studio (Microsoft Corporation, Redmond, WA, USA, v1.82) for the following time intervals considering force onset: 0–50 ms, 0–100 ms, 0–150 ms, 0–200 ms, 50–100 ms, 100–200 ms, and 150–200 ms. The onset of force production was defined by visual detection [12] as previously suggested [22].

### 2.3. Statistical Analyses

Data are presented as mean ± standard deviation. Data analysis was performed using IBM SPSS Statistics 27.0 (IBM Corporation, Armonk, NY, USA). Normality of data distribution was confirmed using the Shapiro–Wilk test. To estimate the test–retest reliability of the IMTP, KF, and SCR exercises, intraclass correlation coefficients (ICC) and the two-way random effects model of the measurements with 95% CI were used [23]. The ICCs were classified as poor (<0.5), moderate (≥0.5–<0.75), good (≥0.75–0.9), and excellent (>0.9) [24]. Wilcoxon r effect sizes were determined and classified as small (r ≤ 0.10–<0.3), moderate effect (r ≥ 0.30–<0.5), and large effect (r ≥ 0.5) for non-normally distributed data based on benchmarks [25]. The standard error of measurement (SEM) and the minimal detectable change (MDC) were calculated to analyze the variability of the participants’ performances. Finally, the within-subject correlations (r) [26] were tested between test and retest for PF and RFD variables. We qualitatively interpreted the magnitudes of correlation using the following criteria: trivial (r ≤ 0.1), small (r = 0.1–0.3), moderate (r = 0.3–0.5), large (r = 0.5–0.7), very large (r = 0.7–0.9), and almost perfect (r ≥ 0.9). Statistical significance was set at *p* < 0.05.

## 3. Results

The results of repeatability and reliability of the IMTP can be seen in Table 1. PF showed excellent reliability (ICC = 0.93) with a very large correlation (r = 0.87; *p* < 0.001). Regarding RFD, most parameters demonstrated moderate reliability (ICC = 0.50–0.63) with moderate correlations (r = 0.34–0.44).

The results of repeatability and reliability for the left and right limbs in the KF exercise can be seen in Table 2 and Table 3, respectively. An excellent reliability was seen for PT (left: ICC = 0.99; right: ICC = 0.97) with almost perfect correlations (left: r = 0.98, *p* < 0.001; right: r = 0.97; *p* < 0.001). Excellent reliability was observed for RTD parameters on the left side (ICC = 0.90–0.96) with very large to almost perfect correlations (r = 0.84–0.93), except for RTD 0–50 ms, which showed poor reliability (ICC = 0.33) with a large correlation (r = 0.58). For the right side, moderate to excellent reliability was seen (ICC = 0.68–0.96) with large to almost perfect correlations (r = 0.51–0.95), except for RTD 0–50 ms, which showed poor reliability (ICC = −0.19) and a trivial correlation (r = 0.09).

The results of repeatability and reliability for the SCR can be seen in Table 4. For the SCR, a good reliability (ICC = 0.75) with a significantly large correlation (r = 0.62; *p* < 0.001) was observed for PF. Finally, poor to moderate reliability was seen for RFD parameters (ICC = 0.14–0.53).

## 4. Discussion

To the best of our knowledge, this is the first study and equipment measuring different types of exercises such as IMTP, KF, and SCR. The main findings were: (1) PF showed excellent repeatability for IMTP and KF exercises with good repeatability for SCR; (2) PF displayed excellent reliability with a very large correlation for IMTP, excellent reliability with an almost perfect correlation for KF, and moderate reliability with a non-significant moderate correlation for SCR; (3) RFD demonstrated moderate to excellent reliability for IMTP, poor to excellent reliability for KF, and poor to moderate reliability for SCR.

The IMTP showed excellent reliability for PF (ICC = 0.93) and a very large within-subject correlation between test and retest (r = 0.87; *p* < 0.001). These results are in accordance with previous studies [9,27,28], including Thomas et al. (2017) and De Witt et al. (2018) reported good reliability for bilateral measures of PF, ICC = 0.86 [28] and ICC = 0.89 [27], respectively. Moreover, Aben et al. (2020) reported excellent reliability when measuring 10 male rugby players (ICC = 0.92) [9]. Regarding RFD, parameters demonstrated moderate reliability (ICC = 0.50–0.63) with moderate correlations (r = 0.34–0.44). These results are not in accordance with previous studies [29,30] which showed good to excellent reliability for RFD parameters at 0–50, 0–100, 0–150, and 0–200 ms. One possible explanation for this discrepancy could be the greater familiarity of rugby players with this type of testing protocol. Indeed, it is known that rugby players perform multiple strength sessions each week [31,32,33] with the deadlift being one of the most common exercises [34,35], which is similar to the IMTP test. In the present study, the participants were physically active males who underwent only a single familiarization session for each test. This interpretation is supported by the good repeatability values observed (ICC = 0.80–0.88), with the exception of the RFD 150–200 ms, which demonstrated moderate reliability (ICC = 0.67).

For the KF exercise, excellent reliability was observed for PT, which is in accordance with the gold standard (isokinetic) equipment [36] and superior to values from a handheld dynamometer [37]. Regarding RTD, most parameters showed excellent reliability, except for RTD 0–50 ms, which was poor for both legs. It should be noted that unilateral KF is not an exercise that individuals are familiar with in their daily lives. This may account for the participants’ difficulty in generating maximal force as rapidly as possible, which likely contributed to the lower ICC observed in the initial interval. It is worth noting that RFD for the hamstrings can be measured with confidence (i.e., ICC  > 0.70 and standard error < 10%) [38]. In the present study, the standard error values exceeding 10% were observed for RTD−0–50 ms (14.8%), RTD 0–100 ms (11.8%), and RTD 150–200 ms (17%). Also, RTD 0–50 ms exhibited an ICC < 0.7. Therefore, it can be concluded that the present device is reliable for PT and RTD parameters specially measured with a minimum window of 100 ms.

For the SCR exercise, excellent repeatability was seen for PF (ICC = 0.94), while RFD parameters were inconsistent, ranging from poor to good repeatability (ICC = 0.23–0.78). Furthermore, good reliability was seen for PF (ICC = 0.75). The RFD parameters also demonstrated inconsistent results ranging from poor to moderate reliability (ICC = 0.14–0.53). Considering the poor to moderate (r = 0.07–0.4) within-subject correlation between test and retest, it is important to highlight that RFD assessments might be challenging and require more time for familiarization with the test [22]. These results partially align with previous studies; using an isokinetic dynamometer, excellent repeatability was reported [15,16]. However, using a hand-held dynamometer for PF measurements, intra-day reliability was poor to moderate (ICC = 0.56 [0.29–0.74]), and inter-tester associations between days were poor (ICC = 0.23 and 0.15). Moreover, a previous study reported poor reliability for peak RTD (ICC = 0.13) [17]. Therefore, it is possible to conclude that the present device has lower repeatability than the gold standard isokinetic machine but higher repeatability and reliability than a hand-dynamometer for plantar flexion.

It should be noted that RFD is sensitive to participant familiarization [22,39,40]. In the present study, only one familiarization session plus two data collection sessions were conducted, which naturally impacted certain RFD parameters. Since variations in participants’ force production can influence the outcomes of equipment analysis, it may be useful to adjust the coefficients of variation across sessions to minimize this impact.

This study is limited by the exercises themselves. Secondly, no direct comparison was made between the current portable dynamometer and gold standard equipment, such as isokinetic dynamometers or other similar portable devices that have already been validated. Despite the high values of repeatability and reliability in PT for KF, the sample size must be taken into consideration, as only 11 participants were able to complete the second session. Additionally, only a single familiarization session was conducted, and potential learning effects across sections must be considered. Given the known sensitivity of RFD to participant familiarization, future studies should consider including a greater number of sessions. Finally, the sampling frequency of the custom-built equipment is 80 Hz, which naturally does not capture as many data points as recommended for RFD analysis. However, the aim of the present study was to effectively assess the equipment’s ability to detect variations in these parameters across different exercises. Despite the limited familiarization, RFD values for the IMTP and KF were satisfactory.

The applicability of the *π*force machine may encompass several domains, such as monitoring fatigue in soccer players throughout the season [2], or identifying athletes’ profiles and their longitudinal development [12]. Additionally, future studies may employ the *π*force to determine baseline values of maximal force production to help decision making within RTP protocols.

## 5. Conclusions

In conclusion, the device that was developed and used in the present study can be effectively employed to assess maximal strength across a variety of resistance exercises, demonstrating consistently good levels of both repeatability and reliability specifically for PF and PT measurements. While the IMTP and KF exercises exhibited moderate to high reliability across the majority of RFD parameters, the SCR exercise showed only poor to moderate levels of repeatability and reliability for most RFD-related outcomes. Consequently, it is recommended that researchers and practitioners exercise a considerable degree of caution when interpreting or analyzing data obtained from SCR assessments, as the variability in measurement may limit the precision and applicability of the findings.

## Figures and Tables

**Table 1:** Repeatability and Reliability analysis for Isometric Mid-Thigh Pull exercise.

Repeatability					Reliability							
	MVC 1	MVC 2	CV (%)	ICC (95% CI)	DAY 1	DAY 2	CV (%)	ICC (95% CI)	SEM	MDC	r	*p*
**Peak Force** (N)	773.1 ± 90.1	753.4 ± 94.1	12%	0.93 [0.86–0.97]	759.6 ± 96.2	782.1 ± 89.9	12%	0.93 [0.85–0.97]	24	68	0.87	<0.001
**RFD 0–50** (N/s)	3159.7 ± 2014.9	3004.6 ± 1787.1	61%	0.88 [0.76–0.94]	3714.8 ± 1598.4	3489.9 ± 1941.6	49%	0.5 [−0.06–0.76]	1253	3469	0.34	0.070
**RFD 0–100** (N/s)	3728.9 ± 1646.9	3495.8 ± 1474.7	43%	0.86 [0.70–0.93]	4090.6 ± 1293.8	3961.0 ± 1412.8	33%	0.60 [0.17–0.81]	855	2369	0.44	0.016
**RFD 0–150** (N/s)	3133.4 ± 1071.9	3000.8 ± 1064.1	34%	0.82 [0.62–0.91]	3382.3 ± 813.7	3312.4 ± 874.9	25%	0.60 [0.16–0.81]	533	1480	0.43	0.018
**RFD 0–200** (N/s)	2629.4 ± 773.0	2513.6 ± 835.7	31%	0.82 [0.62–0.91]	2847.4 ± 565.2	2742.3 ± 646.8	21%	0.63 [0.23–0.83]	373	1034	0.47	0.010
**RFD 50–100** (N/s)	4298.3 ± 1561.6	3986.9 ± 1475.0	36%	0.80 [0.58–0.91]	4598.6 ± 1280.1	4552.6 ± 1286.3	28%	0.57 [0.08–0.79]	846	2342	0.39	0.032
**RFD 100–150** (N/s)	2006.8 ± 1114.6	2011.0 ± 1148.9	56%	0.86 [0.71–0.94]	2346.8 ± 970.5	2305.0 ± 1003.8	42%	0.56 [0.08–0.79]	657	1819	0.39	0.032
**RFD 150–200** (N/s)	1117.2 ± 740.3	1094.7 ± 659.5	63%	0.67 [0.31–0.84]	1570.8 ± 688.4	1359.1 ± 705.2	47%	0.53 [0.03–0.77]	478	1325	0.36	0.053

**Abbreviations:** MVC, Maximal Voluntary Contraction; PF, Peak Force; RFD, Rate of Force Development, at 0–50 ms (RFD 0–50), 0–100 ms (RFD 0–100), 0–150 ms (RFD 0–150), 0–200 ms (RFD 0–200), 50–100 ms (RFD 50–100), 100–150 ms (RFD 100–150), 150–200 ms (RFD 150–200); CV, Coefficient of Variation; ICC, Intraclass Correlation Coefficient; SEM, Standard Error of Measurement; MDC, Minimal Detectable Change; r, Pearson correlation coefficient; *p*, *p* value.

**Table 2:** Repeatability analysis for Knee Flexion exercise.

Left					Right			
	MVC 1	MVC 2	CV (%)	ICC (95% CI)	MVC 1	MVC 2	CV (%)	ICC (95% CI)
**Peak Torque** (N⋅m)	99.45 ± 30.82	102.43 ± 31.09	31%	0.98 [0.93–1.00]	101.46 ± 24.40	106.17 ± 26.76	25%	0.97 [0.86–0.99]
**RTD 0–50** (N⋅m/s)	314.32 ± 117.83	356.06 ± 203.69	50%	0.53 [−0.78–0.88]	404.77 ± 110.98	390.29 ± 174.90	37%	0.61 [−0.59–0.90]
**RTD 0–100** (N⋅m/s)	487.75 ± 153.69	513.84 ± 229.45	39%	0.77 [0.12–0.94]	557.15 ± 114.22	554.14 ± 209.81	30%	0.79 [0.18–0.94]
**RTD 0–150** (N⋅m/s)	458.41 ± 127.99	483.77 ± 173.12	32%	0.87 [0.52–0.96]	496.90 ± 98.39	512.64 ± 172.99	28%	0.89 [0.58–0.97]
**RTD 0–200** (N⋅m/s)	389.97 ± 109.57	410.66 ± 134.88	31%	0.91 [0.65–0.97]	416.70 ± 88.52	435.23 ± 135.40	27%	0.92 [0.72–0.98]
**RTD 50–100** (N⋅m/s)	661.19 ± 203.97	671.62 ± 276.02	36%	0.85 [0.42–0.96]	709.52 ± 154.99	717.98 ± 275.78	31%	0.83 [0.33–0.95]
**RTD 100–150** (N⋅m/s)	399.73 ± 144.46	423.63 ± 146.10	35%	0.81 [0.28–0.95]	376.41 ± 135.45	429.66 ± 159.78	37%	0.83 [0.40–0.95]
**RTD 150–200** (N⋅m/s)	184.65 ± 113.68	191.34 ± 97.41	56%	0.78 [0.13–0.94]	176.10 ± 92.05	203.00 ± 95.62	50%	0.78 [0.23–0.94]

**Abbreviations:** MVC, Maximal Voluntary Contraction; PT, Peak Torque; RTD, Rate of Torque Development, at 0–50 ms (RTD 0–50), 0–100 ms (RTD 0–100), 0–150 ms (RTD 0–150), 0–200 ms (RTD 0–200), 50–100 ms (RTD 50–100), 100–150 ms (RTD 100–150), 150–200 ms (RTD 150–200); CV, Coefficient of Variation; ICC, Intraclass Correlation Coefficient; SEM, Standard Error of Measurement; MDC, Minimal Detectable Change; r, Pearson correlation coefficient; *p*, *p* value.

**Table 3:** Reliability analysis for Knee Flexion exercise.

Left									Right							
	DAY 1	DAY 2	CV (%)	ICC (95% CI)	SEM	MDC	r	*p*	DAY 1	DAY 2	CV (%)	ICC (95% CI)	SEM	MDC	r	*p*
**Peak Torque** (N⋅m)	104.41 ± 31.53	105.61 ± 30.96	30%	0.99 [0.97–1.00]	3.12	8.66	0.98	<0.001	105.55 ± 25.88	108.68 ± 21.52	22%	0.97 [0.89–0.99]	4.12	11.43	0.97	<0.001
**RTD 0–50** (N⋅m/s)	406.22 ± 170.84	369.00 ± 238.36	53%	0.33 [−1.86–0.83]	169.74	470.49	0.59	0.095	447.93 ± 123.53	523.97 ± 186.42	33%	−0.19 [−4.54–0.74]	172.50	478.15	0.10	0.81
**RTD 0–100** (N⋅m/s)	572.86 ± 148.91	604.21 ± 195.89	30%	0.90 [0.57–0.98]	55.02	152.51	0.84	0.046	602.44 ± 125.50	660.14 ± 175.07	24%	0.73 [−0.06–0.94]	79.15	219.38	0.62	0.073
**RTD 0–150** (N⋅m/s)	511.64 ± 120.14	522.39 ± 142.27	25%	0.94 [0.75–0.99]	32.25	89.40	0.90	0.011	527.65 ± 119.59	557.23 ± 116.18	22%	0.92 [0.67–0.98]	33.35	92.43	0.87	0.002
**RTD 0–200** (N⋅m/s)	426.34 ± 110.12	427.46 ± 111.76	26%	0.96 [0.82–0.99]	22.19	61.50	0.91	<0.001	436.37 ± 104.25	454.36 ± 93.52	22%	0.96 [0.81–0.99]	19.81	54.90	0.93	0.003
**RTD 50–100** (N⋅m/s)	745.78 ± 193.08	770.82 ± 237.78	29%	0.93 [0.69–0.98]	57.30	158.84	0.87	0.002	759.27 ± 183.26	805.61 ± 196.03	24%	0.96 [0.76–0.99]	37.95	105.19	0.95	<0.001
**RTD 100–150** (N⋅m/s)	446.83 ± 174.48	428.96 ± 142.05	36%	0.95 [0.81–0.99]	35.57	98.61	0.93	<0.001	426.21 ± 157.59	422.80 ± 91.74	30%	0.64 [−0.92–0.92]	77.36	214.44	0.50	0.17
**RTD 150–200** (N⋅m/s)	214.26 ± 113.69	186.29 ± 119.66	58%	0.93 [0.71–0.98]	30.88	85.59	0.88	0.001	214.04 ± 86.78	186.18 ± 96.13	46%	0.68 [−0.36–0.93]	51.80	143.59	0.51	0.16

**Abbreviations:** PT, Peak Torque; RTD, Rate of Torque Development, at 0–50 ms (RTD 0–50), 0–100 ms (RTD 0–100), 0–150 ms (RTD 0–150), 0–200 ms (RTD 0–200), 50–100 ms (RTD 50–100), 100–150 ms (RTD 100–150), 150–200 ms (RTD 150–200); CV, Coefficient of Variation; ICC, Intraclass Correlation Coefficient; SEM, Standard Error of Measurement; MDC, Minimal Detectable Change; r, Pearson correlation coefficient; *p*, *p* value.

**Table 4:** Repeatability and Reliability analysis for Seated Calf Raise exercise.

Repeatability					Reliability							
	DAY 1	DAY 2	CV (%)	ICC (95% CI)	DAY 1	DAY 2	CV (%)	ICC (95% CI)	SEM	MDC	r	*p*
**Peak Force** (N)	902.2 ± 239.4	911.2 ± 223.8	26%	0.94 [0.87–0.97]	946.9 ± 225.2	900.5 ± 169.9	21%	0.75 [0.48–0.88]	99	274	0.62	<0.001
**RFD 0–50** (N/s)	1426.4 ± 1278.3	1566.8 ± 985.8	76%	0.23 [−0.62–0.63]	2015.3 ± 1195.6	1404.0 ± 1114.0	67%	0.53 [0.002–0.77]	794	2200	0.36	0.053
**RFD 0–100** (N/s)	2187.8 ± 1205.4	2476.6 ± 1259.7	52%	0.32 [−0.42–0.68]	2970.5 ± 1026.8	2002.6 ± 1524.1	52%	0.53 [0.002–0.77]	888	2460	0.38	0.036
**RFD 0–150** (N/s)	2419.2 ± 1062.7	2476.1 ± 1065.6	43%	0.46 [−0.13–0.74]	3109.1 ± 828.9	2009.9 ± 1450.5	45%	0.51 [−0.03–0.77]	820	2272	0.40	0.03
**RFD 0–200** (N/s)	2359.3 ± 847.5	2677.9 ± 851.7	33%	0.65 [0.27–0.83]	2887.8 ± 702.8	1843.6 ± 1269.6	43%	0.46 [−0.13–0.74]	754	2091	0.36	0.054
**RFD 50–100** (N/s)	2949.3 ± 1425.2	3386.5 ± 1569.5	47%	0.38 [−0.30–0.71]	3929.8 ± 1178.9	2611.7 ± 1960.5	49%	0.49 [−0.16–0.73]	1149	3184	0.33	0.078
**RFD 100–150** (N/s)	2881.9 ± 1145.9	3283.8 ± 1116.5	36%	0.77 [0.51–0.89]	3497.9 ± 1023.9	2158.1 ± 1530.2	46%	0.37 [−0.33–0.70]	1026	2845	0.24	0.198
**RFD 150–200** (N/s)	2179.7 ± 1008.6	2487.7 ± 1047.1	44%	0.78 [0.54–0.90]	2708.1 ± 1029.8	1535.7 ± 1153.25	51%	0.14 [−0.81–0.59]	1013	2806	0.07	0.698

**Abbreviations***:* MVC, Maximal Voluntary Contraction; PF, Peak Force; RFD, Rate of Force Development, at 0–50 ms (RFD 0–50), 0–100 ms (RFD 0–100), 0–150 ms (RFD 0–150), 0–200 ms (RFD 0–200), 50–100 ms (RFD 50–100), 100–150 ms (RFD 100–150), 150–200 ms (RFD 150–200); CV, Coefficient of Variation; ICC, Intraclass Correlation Coefficient; SEM, Standard Error of Measurement; MDC, Minimal Detectable Change; r, Pearson correlation coefficient; *p*, *p* value.

## Data Availability

Data is contained within the article or Supplementary Material.

## Supplementary Materials

The following supporting information can be downloaded at https://www.mdpi.com/article/10.3390/muscles4030036/s1.
