# Supplementary material for: πForce—Repeatability and Reliability of Peak Force and Rate of Force Development in a Portable Multi-Exercise Device
Source: Muscles. 2025 Sep 1;4(3):36. doi: 10.3390/muscles4030036 (PMC12452728; doi:10.3390/muscles4030036)
Supplement: Supplementary file 1 [file muscles-04-00036-s001.zip › Supplementary Data/File2_RTD Routine.pdf]

```
import os
import  numpy  as  np
import  pandas  as  pd
import datetime
from scipy.signal import butter, filtfilt
import matplotlib.pyplot as plt
from tkinter import Tk, filedialog, simpledialog, StringVar, OptionMenu, Label, Button, messagebox from
openpyxl import Workbook

click_coords = []  # Global variable to store click coordinates
last_line = None   # Global variable to store the last red line


def find_csv_delimiter(file_path, num_lines=5):
    with open(file_path, 'r') as file:
        lines = [file.readline().strip() for _ in range(num_lines)]

    # Check if semicolon (;) is present in any of the lines for
    line in lines:
        if ';' in line:
            return ';'

    # If semicolon is not found, default to comma (,)
    return ','

def on_move(event):
    if event.inaxes:
        x, y = event.xdata, event.ydata for
        line in ax.lines:
            if line.get_label() == 'vline':
                line.set_xdata([x])  # Convert x to a list
            elif line.get_label() == 'hline':
                line.set_ydata([y])  # Convert y to a list
        plt.draw()

def on_click(event):
    global last_line, vline, hline if
    event.inaxes:
        click_coords.clear()
        click_coords.append((event.xdata, event.ydata))
```

```
        if last_line:
            last_line.remove()  # Remove the previous red line
        last_line = ax.axvline(x=event.xdata, color='r', linestyle='--')  # Draw red line at click

        if vline is None:
            vline = ax.axvline(x=event.xdata, color='r', linestyle='--', label='vline')  # Draw vertical line at click else:
            vline.set_xdata([event.xdata])  # Update vertical line position with a list

        if hline is None:
            hline = ax.axhline(y=event.ydata, color='r', linestyle='--', label='hline')  # Draw horizontal line at click else:
            hline.set_ydata([event.ydata])  # Update horizontal line position with a list

        plt.draw()  # Update the plot

def on_key(event):
    if event.key == 'enter' and len(click_coords) > 0:
        plt.close()  # Close the plot when Enter is pressed


def find_onset(data, threshold, clique):
    start_index = clique - 2
    for i in range(start_index, len(data) - 80):
        if np.abs(data[i] - threshold) == np.min(np.abs(data[start_index:start_index + 80] - threshold)):
            if np.all(data[i:i+80] >= threshold):
                return i
    return clique

def ask_exercise(exercise_var, exercise_list):
    exercise_window = Tk()
    exercise_window.title("Select Exercise")

    label = Label(exercise_window, text="Select the exercise:")
    label.pack(pady=10)

    dropdown = OptionMenu(exercise_window, exercise_var, *exercise_list)
    dropdown.config(width=20, font=('Helvetica', 12)) dropdown.pack(padx=10,
    pady=10)
```

```
    selected_label = Label(exercise_window, text="Selected Exercise: " + exercise_var.get())
    selected_label.pack(pady=10)

    def update_selected_label(*args):
        selected_label.config(text="Selected Exercise: " + exercise_var.get())

    exercise_var.trace("w", update_selected_label)

    def confirm_selection():
        exercise_window.quit()

    confirm_button = Button(exercise_window, text="OK", command=confirm_selection)
    confirm_button.pack(pady=10)

    exercise_window.mainloop()
    exercise_window.destroy()

def ask_calculate_torque():
    response = messagebox.askyesno("Calculate Torque", "Calculate Torque?")
    return response

def get_limb_length(limb_length_path, subject_id):
    delimiter = find_csv_delimiter(limb_length_path)
    limb_data = pd.read_csv(limb_length_path, sep = delimiter, header=None)
    limb_data.columns = ["ID", "LimbLength"]
    leg = limb_data[limb_data["ID"] == subject_id]['LimbLength'].iloc[0]

    if isinstance(leg, str):
        leg = leg.replace(',', '.')
        leg = float(leg)

    return leg



    def main(limb_length_path, players_database_path):
    # Initialize
    print('Abrindo arquivo...')
```

```python
# Create the root window
root = Tk()
root.withdraw()  # Hide the root window

# Ask for exercise type
exercise_list = ['Knee Flexion', 'IMTP', 'Nordic', 'Solear']
exercise_var = StringVar()
exercise_var.set(exercise_list[0])  # Set default value

ask_exercise(exercise_var, exercise_list)

selected_exercise = exercise_var.get()

if selected_exercise == "Knee Flexion":
 # Ask if the user wants to calculate torque
    calculate_torque = ask_calculate_torque()

# Get files from user
root.update()
filenames = filedialog.askopenfilenames(filetypes=[("txt types", "")])
fs = 80  # Hz

# Prepare storage for results
if selected_exercise == "Knee Flexion" and calculate_torque:
    y_label = "Torque"
else:
     y_label = "Force"

measures = [f"Peak_{y_label}", 'RFD050', 'RFD0100', 'RFD0150', 'RFD0200', 'RFD50100', 'RFD100150',
'RFD150200', 'RFDmax', 'RFDmax_Time']
results = {measure: [] for measure in measures}

# Initialize a set to store unique conditions extracted from filenames
unique_conditions = set()

#get folder to save data into
parts = filenames[0].split('/')
index = parts.index(parts[-1])
save_folder = '/'.join(parts[:index])
```

```
# Generate a unique filename based on the current date and time current_datetime =
datetime.datetime.now().strftime("%Y%m%d_%H%M%S") excel_file =
os.path.join(save_folder, f"RFD_results_{current_datetime}.xlsx")

# Create an empty Excel file
wb = Workbook()
wb.save(excel_file)


for filename in filenames:
    # Process filename
    x_name = os.path.splitext(os.path.basename(filename))[0]
    condition = x_name.lower().split("_", 1)[1].upper()
    unique_conditions.add(condition)

# Sort the conditions for consistency file_conditions
= sorted(list(unique_conditions))

# Create file_columns based on file_conditions
files_columns = [f"{condition}_{side}" for condition in file_conditions for side in ['left', 'right']]
all_file_columns = []

for filename in filenames:

    # Process filename
    x_name = os.path.splitext(os.path.basename(filename))[0]
    subject_id = int(x_name.split('_')[0][1:])
    condition = x_name.lower().split("_", 1)[1].upper()
    file_columns = [file for file in files_columns if file.startswith(condition)]
    all_file_columns.append(file_columns)

    # Read and preprocess data
    data = pd.read_csv(filename, sep=",")
    data.drop(columns = "Channel 3", inplace=True) data
    = data*(-1) # inverter sinal
    data.columns = ["RIGHT", "LEFT"]

    # get player name from subject id
    database_players = pd.read_excel(players_database_path)
```

```
    player_name = database_players[database_players["ID"] ==
  subject_id]["Name"].reset_index(drop=True)[0]

    # Determine whether to calculate torque or use force rightectly
    if selected_exercise == 'Knee Flexion' and calculate_torque:
        leg = get_limb_length(limb_length_path, subject_id)

        data_left = data[data.columns[1]] * leg * np.sin(np.deg2rad(90))
        data_right = data[data.columns[0]] * leg * np.sin(np.deg2rad(90))

    else:
        data_left = data[data.columns[1]]
        data_right = data[data.columns[0]]

    # Apply Butterworth filter
    n = 2
    Wn = 7 / (fs / 2)
    b, a = butter(n, Wn, 'low') data_left_filt
    = filtfilt(b, a, data_left) data_right_filt
    = filtfilt(b, a, data_right)

    # Calculate offset
    offset_data_left = np.mean(data_left_filt[4:8])
    offset_data_right = np.mean(data_right_filt[4:8])

    data_left_filt_corr = data_left_filt - offset_data_left
    data_right_filt_corr = data_right_filt - offset_data_right


    global fig, ax, last_line, vline, hline

    # Plot and get user input for onset of left leg fig,
    ax = plt.subplots()
    # Initialize guide lines at the start
    x_initial, y_initial = 0, 0  # Initial cursor position
    vline = ax.axvline(x=x_initial, color='r', linestyle='--', label='vline')  # Draw red vertical line at the start hline
    = ax.axhline(y=y_initial, color='r', linestyle='--', label='hline')  # Draw red horizontal line at the
start
```

```
    ax.plot(data_left_filt_corr, 'b')
    ax.set_title(f'{x_name} - {y_label} membro left')
    ax.set_xlabel('Clique no instante do Onset e pressione Enter para confirmar')
    print('Clique no instante do Onset e pressione Enter para confirmar')

    # Connect the mouse motion, click, and key press events
    fig.canvas.mpl_connect('motion_notify_event', on_move)  # Connect the mouse motion event
    fig.canvas.mpl_connect('button_press_event', on_click)  # Connect the mouse click event
    fig.canvas.mpl_connect('key_press_event', on_key)  # Connect the key press event

    plt.show()

    # Wait for the click coordinates if
    len(click_coords) == 0:
        print("Nenhum onset point selecionado para o membro left. Exiting.")
        return

    onset_click_left = click_coords.pop(0)
    clique = int(onset_click_left[0])

    # Calculate threshold
    Limiar_data_left = onset_click_left[1]

    # Detect onset
    onset_data_left = find_onset(data_left_filt_corr, Limiar_data_left, clique)
    data_left_filt_corr = data_left_filt_corr - data_left_filt_corr[onset_data_left]

    fig, ax = plt.subplots()
    ax.plot(data_left_filt_corr, 'k')
    ax.set_title(f'{x_name} - Onset {y_label} membro left')
    ax.axvline(x=onset_data_left, color='r', linestyle='--') plt.show()
    plt.close()

    # Find peak torques or forces
    Pico_data_left = np.max(data_left_filt_corr[onset_data_left:])
    iPico_data_left = np.argmax(data_left_filt_corr[onset_data_left:]) +
len(data_left_filt_corr[:onset_data_left])
```

```
# Plot and get user input for onset of right leg
fig, ax = plt.subplots()

# Initialize guide lines at the start
x_initial, y_initial = 0, 0  # Initial cursor position
vline = ax.axvline(x=x_initial, color='r', linestyle='--', label='vline')  # Draw red vertical line at the start hline
= ax.axhline(y=y_initial, color='r', linestyle='--', label='hline')  # Draw red horizontal line at the
start


ax.plot(data_right_filt_corr, 'b')
ax.set_title(f'{x_name} - {y_label} membro right')
ax.set_xlabel('Clique no instante do Onset e pressione Enter para confirmar')
print('Clique no instante do Onset e pressione Enter para confirmar')

# Connect the mouse motion, click, and key press events
fig.canvas.mpl_connect('motion_notify_event', on_move)  # Connect the mouse motion event
fig.canvas.mpl_connect('button_press_event', on_click)  # Connect the mouse click event
fig.canvas.mpl_connect('key_press_event', on_key)  # Connect the key press event

plt.show()

# Wait for the click coordinates if
len(click_coords) == 0:
    print("Nenhum onset point selecionado para o membro right. Exiting.")
    return

onset_click_right = click_coords.pop(0)
clique = int(onset_click_right[0])

Limiar_data_right =

onset_click_right[1]

# Detect onset
onset_data_right = find_onset(data_right_filt_corr, Limiar_data_right, clique)
data_right_filt_corr = data_right_filt_corr - data_right_filt_corr[onset_data_right]


fig, ax = plt.subplots()
ax.plot(data_right_filt_corr, 'k')
ax.set_title(f'{x_name} - Onset {y_label} membro right')
ax.axvline(x=onset_data_right, color='r', linestyle='--')
```

```
plt.show()
plt.close()

#Find peak torques or forces
Pico_data_right = np.max(data_right_filt_corr[onset_data_right:])
iPico_data_right = np.argmax(data_right_filt_corr[onset_data_right:]) +
len(data_right_filt_corr[:onset_data_right])

# Calculate RFD
RFD_left = np.diff(data_left_filt_corr[onset_data_left:iPico_data_left]) * (1 / 80)
Pico_RFD_left = np.max(RFD_left)
iPico_RFD_left = np.argmax(RFD_left) / 80

RFD_50_left = (data_left_filt_corr[onset_data_left + 4] - data_left_filt_corr[onset_data_left]) / 0.050
RFD_100_left = (data_left_filt_corr[onset_data_left + 8] - data_left_filt_corr[onset_data_left]) / 0.100
RFD_150_left = (data_left_filt_corr[onset_data_left + 12] - data_left_filt_corr[onset_data_left]) /
0.150
RFD_200_left = (data_left_filt_corr[onset_data_left + 16] - data_left_filt_corr[onset_data_left]) /
0.200
RFD_50_100_left = (data_left_filt_corr[onset_data_left + 8] - data_left_filt_corr[onset_data_left + 4])
/ 0.050
RFD_100_150_left  = (data_left_filt_corr[onset_data_left + 12] - data_left_filt_corr[onset_data_left +
8]) / 0.050
RFD_150_200_left = (data_left_filt_corr[onset_data_left + 16] - data_left_filt_corr[onset_data_left +
12]) / 0.050

RFD_right = np.diff(data_right_filt_corr[onset_data_right:iPico_data_right]) * (1 /
80) Pico_RFD_right = np.max(RFD_right)
iPico_RFD_right = np.argmax(RFD_right) / 80

RFD_50_right = (data_right_filt_corr[onset_data_right + 4] - data_right_filt_corr[onset_data_right]) / 0.050
RFD_100_right = (data_right_filt_corr[onset_data_right + 8] - data_right_filt_corr[onset_data_right]) / 0.100
RFD_150_right = (data_right_filt_corr[onset_data_right + 12] - data_right_filt_corr[onset_data_right]) / 0.150
RFD_200_right = (data_right_filt_corr[onset_data_right + 16] - data_right_filt_corr[onset_data_right]) / 0.200
RFD_50_100_right = (data_right_filt_corr[onset_data_right + 8] - data_right_filt_corr[onset_data_right + 4]) /
0.050
RFD_100_150_right  = (data_right_filt_corr[onset_data_right + 12] - data_right_filt_corr[onset_data_right + 8])
/
0.050
RFD_150_200_right = (data_right_filt_corr[onset_data_right + 16] - data_right_filt_corr[onset_data_right + 12])
/
0.050
```

```
# Append results
results[f"Peak_{y_label}"] = [subject_id, player_name, Pico_data_left, Pico_data_right]
results['RFD050'] = [subject_id, player_name, RFD_50_left, RFD_50_right] results['RFD0100'] =
[subject_id, player_name, RFD_100_left, RFD_100_right] results['RFD0150'] = [subject_id,
player_name, RFD_150_left, RFD_150_right] results['RFD0200'] = [subject_id, player_name,
RFD_200_left, RFD_200_right] results['RFD50100'] = [subject_id, player_name,
RFD_50_100_left, RFD_50_100_right] results['RFD100150'] = [subject_id, player_name,
RFD_100_150_left, RFD_100_150_right] results['RFD150200'] = [subject_id, player_name,
RFD_150_200_left, RFD_150_200_right]


# Open ExcelWriter outside the loop
with pd.ExcelWriter(excel_file, mode='a', if_sheet_exists='overlay') as writer:
    for measure, data in results.items():
        # Prepare DataFrame with all columns
        all_columns = ['Id', 'Name'] + files_columns

        # file new data
        df_columns = ['Id', 'Name'] + file_columns
        df = pd.DataFrame([data], columns = df_columns)

        try:
            # Load existing data from the Excel file
            df_measure = pd.read_excel(writer, sheet_name=measure)

        except ValueError as e:
            if "Worksheet named" in str(e):
                # If the sheet doesn't exist, create it and write the data data
                = [None]*len(all_columns)
                df_measure = pd.DataFrame([data], columns = all_columns)
                df_measure[df_columns] = df
                df_measure["Id"] = df_measure["Id"].astype(int)
                df_measure.to_excel(writer, sheet_name=measure, index=False)
                continue
            else:
                raise e
```

```
            # Initialize DataFrame to hold existing data for the current measure
            existing_data = pd.DataFrame()

            # Merge existing data with new data
            existing_data = df_measure[df_measure['Id'] == df['Id'].iloc[0]]

            # If ID already exists, update the corresponding row with new data if not
            existing_data.empty:
                for col in file_columns:
                    df_measure.loc[df_measure['Id'] == df['Id'].iloc[0], col] = df[col].iloc[0]
            else:
                # If ID doesn't exist, concatenate the new row to df_measure df_measure =
                pd.concat([df_measure, df], axis=0, ignore_index=True)

            df_measure["Id"] = df_measure["Id"].astype(int)
            df_measure.to_excel(writer, sheet_name=measure, index=False)

        print(f"Results for {x_name} saved to {excel_file}")


    with pd.ExcelWriter(excel_file, engine='openpyxl', mode='a') as writer:
        writer.book.remove(writer.book['Sheet'])
    # Print a final message
    print("All files processed.")


if __name__ == '__main__':
    limb_length_csv_path = "Knee Flexion/Limblength.csv"
    players_database = "Base de dados Geral.xlsx"
    main(limb_length_csv_path, players_database)
```
