# Supplementary figures and images for: πForce—Repeatability and Reliability of Peak Force and Rate of Force Development in a Portable Multi-Exercise Device
Source: Muscles. 2025 Sep 1;4(3):36. doi: 10.3390/muscles4030036 (PMC12452728; doi:10.3390/muscles4030036)

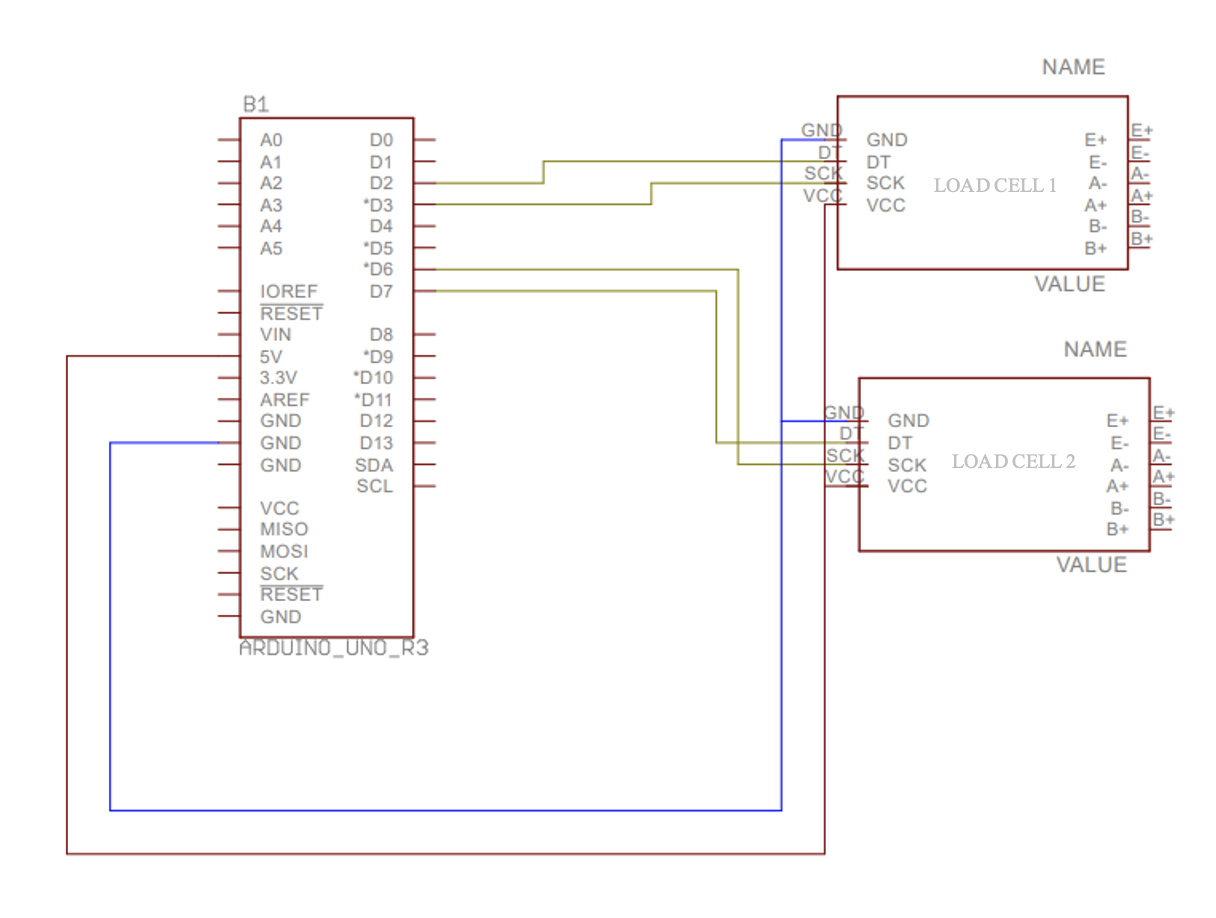

Supplement: Supplementary file 1 [file muscles-04-00036-s001.zip › Supplementary Data/File-1-Diagram.tiff]
